# Generic Delivery of Payload of Nanoparticles Intracellularly via Hybrid Polymer Capsules for Bioimaging Applications

**DOI:** 10.1371/journal.pone.0036195

**Published:** 2012-05-23

**Authors:** Haider Sami, Auhin K. Maparu, Ashok Kumar, Sri Sivakumar

**Affiliations:** 1 Department of Biological Sciences and Bioengineering, Indian Institute of Technology Kanpur, Kanpur, Uttar Pradesh, India; 2 Unit of Excellence on Soft Nanofabrication, Department of Chemical Engineering, Indian Institute of Technology Kanpur, Kanpur, Uttar Pradesh, India; King Abdullah University of Science and Technology, Saudi Arabia

## Abstract

Towards the goal of development of a generic nanomaterial delivery system and delivery of the ‘as prepared’ nanoparticles without ‘further surface modification’ in a generic way, we have fabricated a hybrid polymer capsule as a delivery vehicle in which nanoparticles are loaded within their cavity. To this end, a generic approach to prepare nanomaterials-loaded polyelectrolyte multilayered (PEM) capsules has been reported, where polystyrene sulfonate (PSS)/polyallylamine hydrochloride (PAH) polymer capsules were employed as nano/microreactors to synthesize variety of nanomaterials (metal nanoparticles; lanthanide doped inorganic nanoparticles; gadolinium based nanoparticles, cadmium based nanoparticles; different shapes of nanoparticles; co-loading of two types of nanoparticles) in their hollow cavity. These nanoparticles-loaded capsules were employed to demonstrate generic delivery of payload of nanoparticles intracellularly (HeLa cells), without the need of individual nanoparticle surface modification. Validation of intracellular internalization of nanoparticles-loaded capsules by HeLa cells was ascertained by confocal laser scanning microscopy. The green emission from Tb^3+^ was observed after internalization of LaF_3_:Tb^3+^(5%) nanoparticles-loaded capsules by HeLa cells, which suggests that nanoparticles in hybrid capsules retain their functionality within the cells. *In vitro* cytotoxicity studies of these nanoparticles-loaded capsules showed less/no cytotoxicity in comparison to blank capsules or untreated cells, thus offering a way of evading direct contact of nanoparticles with cells because of the presence of biocompatible polymeric shell of capsules. The proposed hybrid delivery system can be potentially developed to avoid a series of biological barriers and deliver multiple cargoes (both simultaneous and individual delivery) without the need of individual cargo design/modification.

## Introduction

Nanomaterials have attracted a great deal of interest in diverse fields, in particular bioimaging, drug delivery, and biosensing [1]. Different kinds of nanomaterials have been used in a variety of imaging applications such as X-ray computed tomography (noble metal nanoparticles-Au, Ag), near-IR optical imaging (noble metal nanoparticles-Au, Ag), fluorescence based imaging (semiconductor quantum dots, lanthanide-doped nanoparticles), magnetic resonance imaging (Iron and gadolinium based materials) etc [2]. For diagnostic and therapeutic applications in biomedicine, nanoparticles must overcome a series of biological barriers (degradation/aggregation in body fluids, phagocytic clearance by reticulo-endothelial system, crossing of the plasma membrane etc) so as to ultimately perform their desired function [2]. Designing of nanomaterials to provide them with the ability of evading these hurdles and achieving desired localization, is key for exploiting their true potential for biomedical applications. For instance, intracellular delivery of nanoparticles can be achieved by engineering the physical (e.g. size and shape) and surface properties (charge, chemical and biomoities) of the nanoparticles [3]–[5]. However, this may require multiple step processes to achieve the required physical/surface properties of nanoparticles which can lead to aggregation and reduction in the desired properties (e.g. optical, magnetic, electrical, etc) of nanoparticles. Additionally, these multiple step processes can be different for different types of nanoparticles. To circumvent these issues, it is desired to have a generic delivery system to deliver the nanoparticle intracellularly without any individual nanoparticle surface modification. Moreover, the delivery system can serve the purpose of avoiding direct contact of the nanoparticles with the body fluids, and thus avoid degradation/aggregation of nanoparticles and their clearance by phagocytic cells. Fabrication of a generic nanomaterial delivery system encompasses different tasks namely, employment of a generic method to load different kinds of nanomaterials in a delivery vehicle, which in itself is capable/designed of avoiding physiological barriers and using this nanomaterial-loaded vehicle to finally deliver the cargo intracellularly. To this end, we have explored the prospect of loading variety of nanomaterials inside the cavity of polymer capsules and use them as generic nanomaterial delivery systems without individual nanoparticle design/surface modification.

The choice of polymer capsules as nanomaterial delivery vehicles is based on their increasingly potential applications such as drug delivery, biosensing, bioimaging, catalysis and biomedicine [6]–[12]. Additionally, these PEM capsules can be used as multifunctional biovehicle because, their physico-chemical properties (e.g. size, composition, porosity and surface functionality) can be easily tailored and controlled [13]. Integration of nanoparticles with polymer capsules is desirable for multifunctional applications (release of cargo, enhance mechanical properties, etc) [14], [15], and can be achieved by incorporating nanoparticles within the two available compartments in PEM capsules, namely- shell and the cavity. To this end of loading nanoparticles in the shell of polymer capsules, few reports are available on the development of multifunctional PEM capsules which possess optical/magnetic nanoparticles sandwiched between the PEM layers along with the prospect of filling the cavity with desirable cargo [16]–[19]. Koo *et al.* have reported loading of gold nanorods on the surface of polymer capsules [20]. However, this approach of loading of ‘pre-synthesized nanoparticles’ within the shell has limitations such as lesser loading of nanoparticles [21], possible release of nanoparticles before reaching the targeted site due to disruption of few layers of polymers, and change in the property of nanoparticles [22]. Recently, Caruso and his coworkers have shown the synthesis of magnetic nanoparticles/QDs-loaded PEM capsules by using preformed nanoparticles in the emulsion template [23]. Even loading of pre-synthesized nanoparticles in template may also lead to change in property of nanoparticles as the nanoparticles will be exposed to the process of capsule synthesis (e.g. emulsification etc) and template removal. To circumvent these issues of using ‘pre-synthesized nanoparticles’ either in the shell or cavity, the nanoparticles can be loaded inside the cavity of polymer capsules, by synthesizing them within the capsule interior. Shchukin *et al.* have reported synthesis of YF_3_ (for yttrium recovery from aqueous solutions) and rare earth phosphates nanoparticles in polyelectrolyte capsules [24], [25]. However, to our knowledge, there is no report available on a general approach to ‘synthesize’ variety of nanomaterials inside the ‘cavity’ of polymer capsules and generic delivery of nanoparticles via polymer capsules.

The proposed work attempts to address the challenge of fabrication of generic nanomaterial delivery system by dealing with it at two different steps. Firstly, we have employed a general and versatile method to synthesize variety of nanomaterials inside the cavity of polymer capsules by templating polystyrene sulfonate (PSS)/polyallylamine hydrochloride (PAH) and cross-linked PAH polymer capsules as micro/nanoreactors. Secondly, nanoparticles-loaded capsules were interacted with human cervical cancer cells (HeLa) to deliver the ‘as prepared’ nanoparticles in a functional state intracellularly, without needing individual nanoparticle design/surface modification. In this report, the microvolume of polymer capsules was exploited to synthesize gold, silver, cadmium sulfide and lanthanide ion-doped nanoparticles (LaF_3_:Tb^3+^(5%), LaVO_4_:Eu^3+^(5%), GdF_3_:Tb^3+^(5%)), stabilized with citrate ligand inside the PSS/PAH capsule. In addition, different shapes of gold nanostructures (nanorods, nanoprisms, and multifaceted nanostructures) and co-loading of two types of nanoparticles within the same polymer capsule have been demonstrated. We have also demonstrated the co-loading of protein (RITC-BSA; Rhodamine B isothiocyanate-bovine serum albumin) along with gold nanoparticles for potential stimuli-responsive (e.g. laser) drug delivery applications. Furthermore, different sizes of polymer capsules (400 nm, 1 µm, and 5 µm) have been used as nano/microreactors to synthesize gold nanoparticles and to prove the generality and versatility of our method. Interaction of these nanoparticles-loaded PSS/PAH capsules with HeLa cells was examined for uptake kinetics and i*n vitro* cytotoxicity studies, thereby demonstrating a generic platform for delivery of a variety of nanoparticles to cells. We have demonstrated synthesis of a) metal nanoparticles (Au and Ag) in capsules as candidates for potential micro-CT imaging applications and laser induced release of cargo, b) lanthanide ion doped inorganic nanoparticles (LaF_3_:Tb^3+^, LaVO_4_:Eu^3+^ and GdF_3_:Tb^3+^) and CdS nanoparticles as candidates for fluorescence based imaging applications and c) GdF_3_ nanoparticles as MRI contrast agents. One of the nanoparticles-loaded capsules was used to demonstrate fluorescence based imaging in HeLa cells *in vitro.*

This approach has several advantages from the standpoint of both generic synthesis and generic delivery of nanomaterials: (i) this method can be applied to load variety of nanoparticles (e.g. Au, Ag, CdS, LaF_3_, GdF_3_, LaVO_4_, etc.) inside the polymer capsule along with loading of therapeutic molecules (e.g. RITC-BSA); (ii) different kinds of nanoparticles can be simultaneously loaded inside the capsule (e.g. Au and LaVO_4_); (iii) different shapes of nanomaterials can be loaded (e.g. gold nanorods, nanoprisms, multifaceted nanostructures) (iv) size, composition, and morphology of capsules/nanoparticles can be easily tailored and controlled; (v) the number of nanoparticles inside the polymer capsules can be easily controlled by varying the concentration of nanoparticle precursors; (vi) variety of nanoparticles can be delivered intracellularly without individual nanoparticle surface modification (e.g. ‘as prepared’ Au and LaF_3_:Tb^3+^ nanoparticles were delivered intracellularly to HeLa cells via polymer capsules); (viii) prevention of nanoparticle exposure to body fluids, avoidance of any nanoparticle release/degradation/change in property before reaching the target, evading direct contact of nanoparticles with cells and generic delivery of a payload of nanoparticles without the step of nanomaterial surface functionalization/design (of every single nanoparticle; required for all types of nanoparticles and is different for different types of nanoparticles) needed for delivery and avoidance of biological barriers.

These hybrid polymer capsules can be potentially used as active targeting (surface modification with ligands/antibodies) as well as passive targeting (based on their size, ∼300–700 nm) [26], [27]. Moreover, these nanomaterials-loaded PEM capsules can be potentially used as multimodal bioimaging agents [28], [29] (magnetic resonance imaging (MRI), X-ray computed tomography (CT), fluorescence imaging, and nuclear imaging), drug delivery vehicles and biosensors. Loading of multiple cargoes (simultaneous loading of two types of nanoparticles/simultaneous loading of nanoparticles and therapeutics) within the capsule can be explored to design multifunctional vehicles for biomedical applications [30]. In addition, the residence time of these nanoparticles-loaded polymer capsules can potentially be longer in the affected tissues compared to bare nanoparticles (size <20 nm) which can facilitate better imaging of affected tissues. One has to bear in mind that the residence time of nanoparticles in the affected tissues depends on their size, shape, and surface [27].

## Results and Discussion

### Generic Method to Prepare Nanoparticles-Loaded Capsules

**Figure 1:**
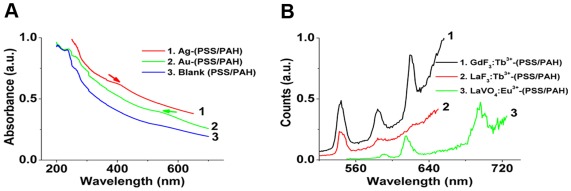
Optical properties of nanoparticles-loaded PSS/PAH capsules. (a) UV-Vis absorbance spectra of (1) Ag and (2) Au nanoparticles-loaded PSS/PAH capsules and (3) blank PSS/PAH capsules; (b) photoluminescence emission spectra of (1) GdF_3_:Tb^3+^(5%), (2) LaF_3_:Tb^3+^(5%) and (3) LaVO_4_:Eu^3+^(5%) nanoparticles-loaded PSS/PAH capsules [Excitation wavelength used was 488 nm for GdF_3_:Tb^3+^(5%) and LaF_3_:Tb^3+^(5%) and 465 nm for LaVO_4_:Eu^3+^(5%)].

Scheme S1 shows the schematic representation of synthesis of nanomaterials-loaded polymer capsules. The PSS/PAH capsules were formed via layer-by-layer (LbL) assembly of polymers on monodisperse silica particles (size ∼5 µm) followed by removal of the core by etching with HF. Further, these capsules were incubated in nanoparticle precursor salts solution along with citrate ions as ligand. The excess nanoparticles formed outside the polymer capsules were removed by washing thrice with water. All the nanoparticles-loaded polymer capsules were easily dispersible in PBS buffer (pH∼7.2) as can be observed from the digital image (Scheme S1 inset). Figure 1a demonstrates the UV-Vis absorption spectra of Au and Ag nanoparticles-loaded PSS/PAH capsules. The appearance of absorbance peaks due to surface plasmon resonance at ∼524 nm (green curve) and ∼400 nm (red curve) suggest the formation of gold and silver nanoparticles, respectively. Furthermore, the absorbance of nanoparicles-loaded capsules matches with the absorbance of blank nanoparticles (Figure S1). Figure 1b shows the photoluminescence (PL) emission spectra of LaF_3_:Tb^3+^(5%), LaVO_4_:Eu^3+^(5%), and GdF_3_:Tb^3+^(5%) nanoparticles-loaded PSS/PAH capsules by excitation with laser. The emission bands (black and red curve, Figure 1b) around 544, 584 and 619 nm are assigned to ^5^D_4_ to ^7^F_5_, ^7^F_4_, and ^7^F_3_ transitions, respectively of Tb^3+^ ions. In addition, the average life time of Tb^3+^ ions in LaF_3_ and GdF_3_ are 1.7 ms and 2.8 ms, respectively, which clearly suggests that the Tb^3+^ ions are doped in an inorganic matrix.[31] The emission bands around 591 nm (^5^D_0_ to ^7^F_1_), 615 nm (^5^D_0_ to ^7^F_2_), and 696 nm (^5^D_0_ to ^7^F_4_) are assigned to Eu^3+^ ions. Additionally, the average lifetime of Eu^3+^ is reported to be 5.3 ms. (Table S2 and Figure S2) [31]. We note that, the background emission in PL emission spectra arises from blank PSS/PAH capsules (Figure S3). Furthermore, the optical properties of the nanoparticles-loaded capsules match with the blank nanoparticles (Figure S4), which clearly suggested that properties of nanoparticles do not change when trapped inside the polymer capsule. Over all, the nanoparticles-loaded PSS/PAH capsules show excellent optical properties warranting their potential for various bioimaging applications.

**Figure 2:**
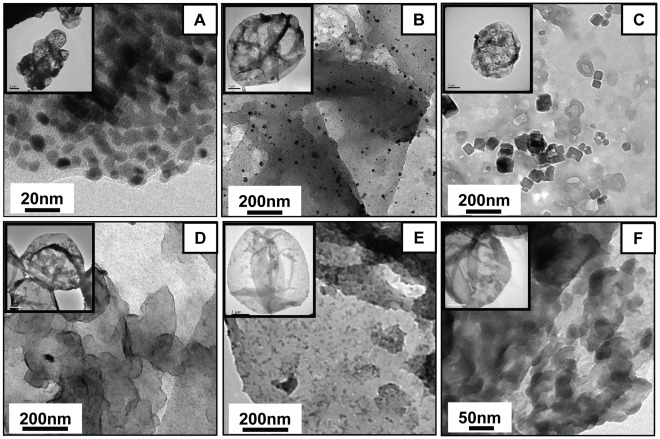
Validation of nanoparticle presence inside the PSS/PAH capsules. Representative TEM image of (a) Au, (b) Ag, (c) LaVO_4_:Eu^3+^(5%), (d) LaF_3_:Tb^3+^(5%), (e) CdS, and (f) GdF_3_:Tb^3+^(5%) nanoparticles-loaded PSS/PAH capsules. (Inset in each figure shows the respective nanoparticles-loaded PSS/PAH capsule at lower magnification.).

**Figure 3:**
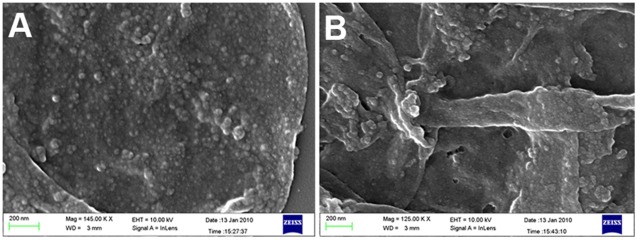
Surface characterization of nanoparticles-loaded PSS/PAH capsules. Scanning electron micrograph of (a) Au nanoparticles-loaded PSS/PAH capsules and (b) blank PSS/PAH capsules.

The size and surface morphology of nanoparticles-loaded PSS/PAH capsules have been characterized by transmission electron microscopy (TEM) and scanning electron microscopy (SEM). Figure 2 demonstrates the TEM image of Au, Ag, LaVO_4_:Eu^3+^(5%), LaF_3_:Tb^3+^(5%), CdS, and GdF_3_:Tb^3+^(5%) nanoparticles-loaded PSS/PAH capsules. The contrast in the TEM image clearly shows that the interior of the polymer capsules are loaded with nanoparticles and the size/shape of the nanoparticles are uniform. As can be observed in the TEM images, the cavity has more contrast when compared to the walls of the capsule, which clearly suggests that the nanoparticles are inside the polymer capsules (if the particles were in the wall, the contrast of the wall of the polymer capsules would have been more as compared to cavity). High loading of nanoparticles can be observed in figure 2a and 2b, showing gold and silver nanoparticles in the size range of 5–10 nm and 5–15 nm respectively. We note that the aggregation observed in the TEM images is due to the drying effect during the sample preparation. To validate the formation of nanoparticles inside polymer capsules, the nanoparticles-loaded polymer capsules were subjected to SEM analysis. The surface morphology of blank capsules and gold nanoparticles-loaded polymer capsules are similar, which clearly suggests that nanoparticles are not on the surface of the PEM capsules (Figure 3). The synthesis of different kinds of nanoparticles inside the polymer capsule clearly proves the generality and versatility of our approach.

**Figure 4:**
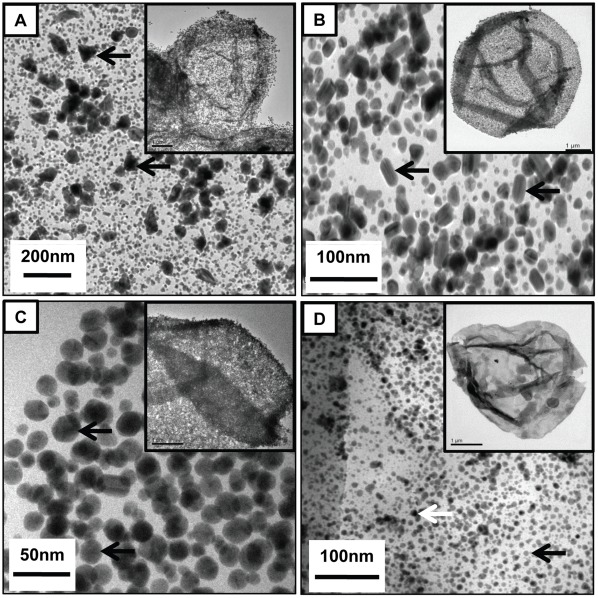
PSS/PAH capsules loaded with different shapes of nanoparticles and co-loaded with two types of nanoparticles. TEM image of PSS/PAH capsules loaded with (a) gold nanoprism, (b) gold nanorods, (c) multifaceted gold nanostructures and (d) Au and LaVO_4_:Eu^3+^(5%) nanoparticles (co-loaded, black and white arrow show LaVO_4_:Eu^3+^(5%) and Au nanoparticles respectively.).

Figure 4a, 4b and 4c shows TEM images of PSS/PAH capsules loaded with different shapes of gold nanostructures, namely gold nanoprisms, gold nanorods and multifaceted gold nanostructures respectively. Figure 4b and 4c suggests that the formation of gold nanorods and multifaceted nanostructures inside the polymer capsule. It was also observed that the capsules possess seed gold particles along with gold nanoprisms. We note that Murphy *et al*. have reported that the synthesis of uniform size and shape of gold nanorods is a challenging process; however, they have improved the yield of nanorods by centrifugation processes [32]. Selective synthesis of different shapes of nanostructures by templating polymer capsules will be further investigated to evade the separation steps. This shows the versatility of the approach in terms of control on shape and adaptability of seed mediated growth of nanoparticles within the capsule. Figure S5a and S5b shows the UV-Vis absorption spectra of PSS/PAH capsules loaded with gold nanorods and multifaceted gold nanostructures respectively. The absorbance in the NIR region further suggests the formation of nanorods and multifaceted nanostructures. This clearly suggests that these capsules loaded with different shapes of nanomaterials can further widen their applications such as stimuli (near infra red (NIR)) responsive drug delivery, photothermal ablation therapy, etc [16], [33].

**Figure 5:**
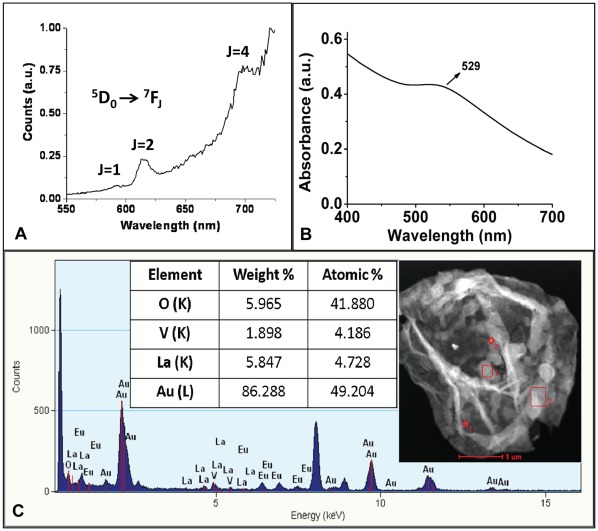
Optical properties of PSS/PAH capsules co-loaded with two types of nanoparticles. (a) Photoluminescence emission spectrum, (b) UV-Vis absorption spectrum and (c) EDX analysis of PSS/PAH capsules co-loaded with Au and LaVO_4_:Eu^3+^(5%) nanoparticles.

Co-loading of two types of nanoparticles inside PSS/PAH capsules was done by loading the capsules with LaVO_4_:Eu^3+^(5%) nanoparticles first and then using these LaVO_4_:Eu^3+^(5%) nanoparticles-loaded capsules for synthesis of gold nanoparticles. As can be observed from TEM image (Figure 4d), the polymer capsules were successfully loaded with two types of particles having different electron densities. The lighter particles (marked with black arrow) are LaVO_4_:Eu^3+^(5%) nanoparticles and gold nanoparticles are the darker particles (marked with white colored arrow). Figure 5c shows the energy-dispersive x-ray (EDX) spectroscopy of Au and LaVO_4_:Eu^3+^-loaded polymer capsules which indicates the presence of La and Au. This further confirms the co-loading of LaVO_4_:Eu^3+^(5%) and gold nanoparticles inside the PSS/PAH capsules. The PSS/PAH capsules co-loaded with LaVO_4_:Eu^3+^(5%) and gold nanoparticles were further investigated for their optical properties to ensure the functionality of the two types of nanoparticles within the capsule interior. Figure 5a shows the photoluminescence emission spectrum of PSS/PAH capsules co-loaded with LaVO_4_:Eu^3+^(5%) and gold nanoparticles, depicting the presence of emission bands around 591 nm (^5^D_0_ to ^7^F_1_), 615 nm (^5^D_0_ to ^7^F_2_), and 696 nm (^5^D_0_ to ^7^F_4_), which are assigned to Eu^3+^ ions. The absorbance peak at 529 nm in Figure 5b indicates the formation of gold nanoparticles in the co-loaded capsules. We note that the life time of Eu^3+^ ions (Table S2) is less in co-loaded sample (LaVO_4_:Eu^3+^(5%) and Au; 1.6 ms) when compared to LaVO_4_:Eu^3+^(5%)(5.3 ms)-loaded polymer capsule. We depict that the reduction in life time may be due to reduction in sizes of LaVO_4_ nanoparticles.

**Figure 6:**
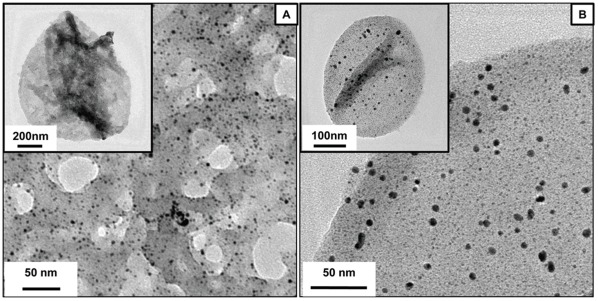
Nanoparticles in different sizes of capsules. TEM image of Au nanoparticles loaded (a) 1 µm sized PSS/PAH capsules and (b) cross-linked PAH nanocapsules (∼400 nm).

To demonstrate further the generality of our method three different sized polymer capsules were employed for synthesis of gold nanoparticles, namely 5 µm PSS/PAH capsules, 1 µm PSS/PAH capsules and cross-linked PAH nanocapsules (∼ 400 nm). Gold nanoparticles were successfully synthesized inside all the three types of capsules as can be observed in Figure 2a, 6a and 6b. The PSS/PAH capsules were co-loaded with RITC-BSA (model drug) and Au nanoparticles to exhibit the multifunctionality of the proposed hybrid materials. To confirm the loading of RITC-BSA, the loaded capsules were subjected to fluorescence microscopy (Figure S6), where the red emission corresponds to the presence of RITC-BSA in the interior of the polymer capsule. These RITC-BSA loaded capsules were then used as templates to synthesize Au nanoparticles by the same method as described above.

Versatility and generality of the approach was emphasized by synthesizing different shaped nanoparticles (nanorods, nanoprisms, and multifaceted nanostructures), co-loading of two types of nanoparticles and co-loading of protein along with Au nanoparticles inside PSS/PAH capsules. Nanoparticle loading inside these capsules was shown by employment of a general procedure using citrate as ligand for all the nanoparticles loaded (Au, Ag, CdS, LaF_3_, GdF_3_, LaVO_4_). Loading of gold nanoparticles inside different sized capsules (PSS/PAH capsules [5 µm, 1 µm] and cross-linked PAH nanocapsules [∼400 nm]) further highlights the generality of our approach.

### Delivery of Nanoparticles Payload to Cancer Cells

**Figure 7:**
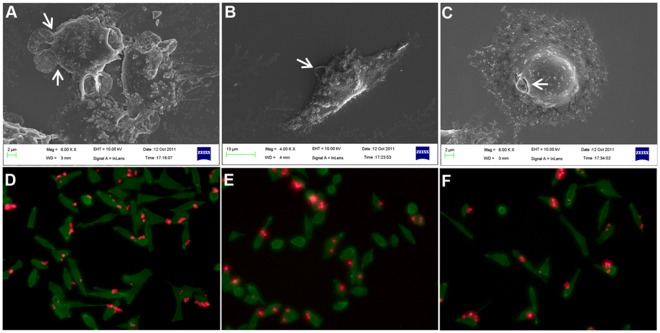
Kinetics of uptake of nanoparticles-loaded capsules by HeLa cells. Cell uptake kinetics of nanoparticles-loaded PSS/PAH capsules by HeLa cells followed by scanning electron microscopy (a–c) and fluorescence microscopy (d–f) as a function of time 2 h (a and d), 5 h (b and e) and 8 h (c and f). To visualize the uptake by fluorescence microscopy, LaF_3_:Tb^3+^(5%) nanoparticles were loaded in RITC-labeled PSS/PAH capsules (red emission) and actin cytoskeleton of cells was stained with FITC-Phalloidin (green). In SEM micrographs, nanoparticles-loaded capsules are indicated by white arrows.

**Figure 8:**
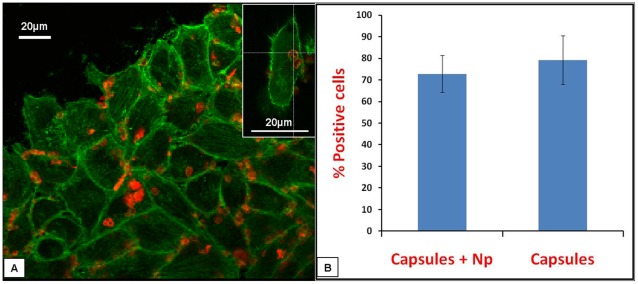
Uptake of nanoparticles-loaded PSS/PAH capsules by HeLa cells. (**a**) Confocal laser scanning microscopy (CLSM) image of HeLa cells after uptake of LaF_3_:Tb^3+^(5%) nanoparticles-loaded PSS/PAH capsule. Inset shows high magnification image of a HeLa cell, which has internalized Au nanoparticles-loaded capsule. Actin cytoskeleton of cells was stained with FITC-Phalloidin (green). The outer PAH layer of the capsules is labeled with RITC (red) and (**b**) efficiency of uptake of Au nanoparticles-loaded PSS/PAH capsules and blank PSS/PAH capsules by HeLa cells as investigated by flow cytometry. Efficiency of uptake is represented by percentage positive cells i.e. cells associated (both internalized and bound) with capsules.

In order to investigate the delivery capabilities of these nanoparticles-loaded capsules, they were subjected to interaction with HeLa cells. Figure 7 shows the uptake kinetics of LaF_3_:Tb^3+^(5%) nanoparticles-loaded PSS/PAH capsules as a function of incubation time with HeLa cells, where the uptake was followed by fluorescence microscopy and scanning electron microscopy. To visualize the uptake by fluorescence microscopy, LaF_3_:Tb^3+^(5%) nanoparticles were loaded in RITC-labeled capsules (red emission) so as to locate the nanoparticles-loaded capsules during the internalization process. The uptake of nanoparticles-loaded capsules by HeLa cells has started around 2–5 hours as binding events [binding of nanoparticles-loaded RITC-labeled capsules (red) to cells (green)] can be observed (Figure 7a, 7b, 7d and 7e) during that durations. Moreover, internalization events can be observed around 8 hours (Figure 7c and 7f) suggesting that the internalization process has initiated around 2–5 hours and is probably near completion around 8 hours of treatment. Further, uptake of LaF_3_:Tb^3+^(5%) and Au nanoparticles-loaded capsules by HeLa cells was visualized by confocal laser scanning microscopy (CLSM) after 16 hours of incubation, where it can be observed that most of the cells (green emission) have internalized the nanoparticles-loaded capsules (red emission from RITC of capsule) (Figure 8a). Moreover, it can also be seen that the internalized capsules are collapsed (Figure 8a inset) suggesting their uptake (also observed in other reports) [34]. CLSM imaging of various Z stacks of cells (Figure S7) after the incubation with nanoparticles-loaded capsules clearly suggests that the nanoparticles-loaded polymer capsules were efficiently internalized by the cells, thus delivering the payload of nanoparticles to the cells. Efficiency of uptake of Au nanoparticles-loaded capsules and blank PSS/PAH capsules by HeLa cells was investigated by flow cytometry to inspect differences in uptake. There was no significant difference in efficiency of uptake of nanoparticles-loaded capsules and blank capsules by HeLa cells as suggested by the FACS analysis (Figure 8b), where the average percentage positive cells was 72.85% and 79.25% for nanoparticles-loaded capsules and blank capsules respectively. This suggests that the process of loading of nanoparticles inside these PSS/PAH capsules does not affect the properties of the capsules required for uptake by the cells. The percentage positive cells were estimated from the 2D dot plots of the events corresponding to association/uptake of the nanoparticles-loaded polymer capsules by the HeLa cells (Figure S8). Events with high forward scattering and high fluorescence intensity were assigned to capsules/nanoparticles-loaded capsules bound/internalized with the cells. Events with low forward scattering and high fluorescence intensity correspond to free capsules/nanoparticles-loaded capsules. Only cells show high forward scattering with low fluorescence intensity.

**Figure 9:**
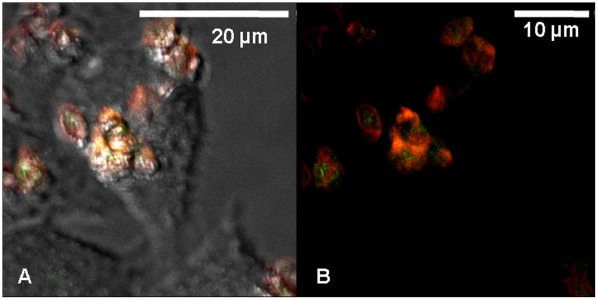
CLSM images showing the characteristic emission of Tb^3+^ from the HeLa cell internalized LaF_3_:Tb^3+^(5%) nanoparticles-loaded PSS/RITC-PAH capsule. (a) Composite image of bright field and fluorescent signals arising from LaF_3_:Tb^3+^(5%) nanoparticles (green emission from Tb^3+^) and RITC-PAH (red); and (b) composite image of fluorescent signals arising from LaF_3_:Tb^3+^(5%) nanoparticles (characteristic green emission from Tb^3+^) and RITC-PAH (red) showing internalized LaF_3_:Tb^3+^(5%) nanoparticles-loaded capsules.

Post uptake, examination of functionality of the nanoparticles is imperative from application point of view. Investigation of emission from Tb^3+^ from the internalized LaF_3_:Tb^3+^(5%) nanoparticles-loaded PSS/PAH capsules by HeLa cell was needed, to assess the optical properties of the nanoparticles post uptake process and while inside the cell. HeLa cells were imaged by CLSM post uptake of LaF_3_:Tb^3+^(5%) nanoparticles-loaded PSS/RITC-PAH capsules and the characteristic green emission from Tb^3+^ was observed (Figure 9) confirming the presence of Tb^3+^ doped nanoparticles in a functional state inside the cell. The green emission (from Tb^3+^) localized with the red emission from RITC-PAH of the polymer wall of the capsules thus confirming the presence of nanoparticles inside the capsules in which they were loaded.

**Figure 10:**
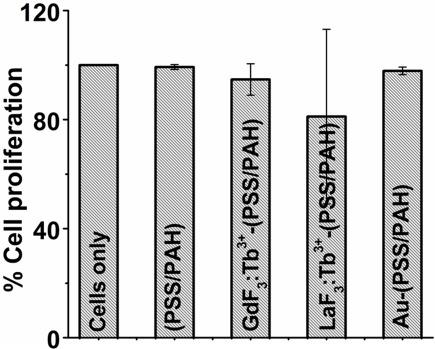
*In vitro* biocompatibility of nanoparticles-loaded PSS/PAH capsules. MTT assay results of HeLa cells, blank PSS/PAH capsules, GdF_3_:Tb^3+^(5%), LaF_3_:Tb^3+^(5%), and Au nanoparticles-loaded PSS/PAH capsules on incubation with HeLa cells.

*In vitro* cytotoxicity of LaF_3_:Tb^3+^ (5%), GdF_3_:Tb^3+^ (5%), and Au nanoparticles-loaded PSS/PAH capsules was studied on HeLa cells by MTT assay, so as to investigate the effect of internalization of these nanoparticles-loaded capsules on cell viability. It was observed that cell proliferation percentages for Au, LaF_3_:Tb^3+^(5%) and GdF_3_:Tb^3+^(5%) nanoparticles-loaded polymer capsules were nearly similar to that of non-treated cell control and blank capsules (Figure 10). It clearly suggests that nanoparticles-loaded polymer capsules show less/no cytotoxicity on HeLa cells, thus confirming their biocompatibility.

Thus a generic way of delivering Au nanoparticles and LaF_3_:Tb^3+^(5%) nanoparticles to HeLa cells was shown via PSS/PAH capsules and validated by CLSM and flow cytometry. We note that both the types of nanoparticles have been delivered intracellularly without the need of any surface modification for individual nanoparticles. Furthermore, this strategy can be extended for delivery of other nanoparticles also, as the method of loading nanoparticles inside the capsules employs a generic approach. Additionally, the fluorescence emission from Tb^3+^ was observed even after the uptake of Tb^3+^ doped nanoparticles-loaded PSS/PAH capsules by the cell, indicating the presence of nanoparticles in their functional state inside the cell and showing their potential as bioimaging agents. Further it was observed that these nanoparticles-loaded capsules show less or no cytotoxicity to the cells post uptake (in comparison to blank capsules or untreated cells), suggesting their *in vitro* compatibility with biological systems.

In conclusion, a general and facile delivery approach to deliver variety of nanoparticles to cells without individual nanoparticles surface modification has been demonstrated. Loading of different kinds of nanoparticles (Au, Ag, CdS, LaF_3_:Tb^3+^, GdF_3_:Tb^3+^, LaVO_4_:Eu^3+^) inside the polymer capsules supports the generality and versatility of the method. Additionally, different morphology of gold nanostructures (nanorods, nanoprisms and multifaceted nanostructures) and co-loading of Au and LaVO_4_:Eu^3+^ nanoparticles further demonstrates the versatility of our approach. Delivery of LaF_3_:Tb^3+^ and Au nanoparticles to HeLa cells thorough polymer capsules as a carrier have been shown. This clearly suggests that this approach can be used as a general method to deliver a payload of different types of nanoparticle without the need of individual nanoparticle surface modification. Furthermore, these nanoparticles-loaded polymer capsules show high biocompatibility and efficient internalization within the cells. Green fluorescence emission from internalized LaF_3_:Tb^3+^nanoparticle-loaded polymer capsules in HeLa cells demonstrates their potential use in bioimaging applications. These nanomaterials-loaded capsules can have potential applications in multimodal bioimaging and drug delivery.

## Materials and Methods

### Materials

All the lanthanide salts, poly-(sodium 4-styrene sulfonate) (PSS, M_w_ 70 kDa), poly-(allylamine hydrochloride) (PAH, M_w_ 70 kDa), FITC-phalloidin (Fluorescein isothiocyanate-phalloidin), trypsin-EDTA, dulbecco’s modified eagle’s medium (DMEM), penicillin-streptomycin antibiotic, (3-(4,5-Dimethylthiazol-2-yl)-2,5-diphenyltetrazolium bromide (MTT), and gelatin (from cold water fish skin) were purchased from Sigma Aldrich and used without further purification. Sodium borohydride and dimethyl sulphoxide were obtained from Merck’s chemicals, India. Fetal bovine serum and chloroauric acid were bought from Hyclone (UT, USA) and LOBA Chemie, India respectively. Hydrofluoric acid, CTAB and cadmium nitrate were obtained from S.D. fine chem. Ltd. Silver nitrate, sodium sulfide, sodium chloride, liquor ammonia, triethylamine, and methanol were purchased from Fisher Scientific. 3-aminopropyltriethoxysilane (APTES) and tri sodium citrate dihydrate was obtained from Spectrochem Pvt. Ltd and RFCL Ltd. respectively. Citric acid and sodium fluoride were purchased from Qualigens. Mesoporous silica particles (5 µm) were purchased from Tessek (Czech Republic). 1 µm sized silica particles were purchased from microparticles GmbH (Berlin, Germany). APTES modified mesoporous silica particles were prepared as described elsewhere [35]. FITC-PAH (Fluorescein isothiocyanate-PAH) and RITC-PAH (Rhodamine B isothiocyanate-PAH) was obtained by labeling PAH with FITC and RITC respectively [36]. HeLa cells were purchased from National Centre for Cell Science Pune, India which is a national repository of cell lines in India.[/LOOSER]

### Preparation of PSS/PAH Capsules

PSS/PAH capsules were prepared as described in detail elsewhere [37]. Briefly, APTES-modified mesoporous silica (10 mg, 5 µm in size) were incubated with PSS and PAH (1 mg/ml of 0.5 M NaCl) solutions alternatively for 15 min to build-up the layers followed by washing thrice with water. Typically, five bilayers were developed and the silica core was removed by treating with 1 ml of 5 M HF for 2 minutes to obtain the PSS/PAH capsules (*Caution: HF is very toxic, so should be handled with all safety precautions*). The capsules were isolated via centrifugation/redispersion cycles (in water). Synthesis of 1 µm sized PSS/PAH capsules were done in a similar by templating 1 µm sized silica particles.

### Synthesis of Cross-linked PAH Nanocapsules

For synthesis of cross-linked PAH nanocapsules, SC/MS (solid core mesoporous shell) silica template particles were synthesized as reported [38]. PAH nanocapsules were prepared as described by Wang *et al* [39]. In brief, the SC/MS template silica particles were taken and polymer was infiltrated in the mesoporous shell by incubating the template particles with PAH solution (5 mg/ml in 0.2 M NaCl; pH 8.5) for overnight with gentle mixing. Post infiltration, the unbound polymer was washed off thrice in water by centrifugation. The polymer was then cross-linked by incubating the polymer-infiltrated particles with glutaraldehyde for 20 min. Finally, the particles were washed with water and the template was etched out by incubating them with 2 M HF: 8 M NH_4_F (pH 5) to get PAH capsules (*Caution: HF is very toxic, so should be handled with all safety precautions*).

### Synthesis of Nanoparticles-loaded Polymer Capsules and Bare Nanoparticles

The PSS/PAH capsules were mixed with stock solution A for 15 min, followed by the incubation with the stock solution B (15 min). Stock solution C was added to the above and the mixture was kept under shaking for 1 h at ambient temperature. Excess nanoparticles formed outside the capsules were removed out by repeated centrifugation and washing steps with water. This procedure was followed to prepare Au, Ag, CdS, LaF_3_:Tb^3+^, GdF_3_:Tb^3+^, and LaVO_4_:Eu^3+^ nanoparticles-loaded PSS/PAH capsules by taking the respective stock solution (Table S1). Blank nanoparticles were synthesized by using the above procedure without templating the capsules. Similar steps were followed to synthesize gold nanoparticles in 1 µm sized PSS/PAH capsules and cross-linked PAH nanocapsules.

### Synthesis of PSS/PAH Capsules-loaded with Gold Nanorods, Gold Nanoprisms and Multifaceted Gold Nanostructures

Gold nanostructures of various shapes were formed inside PSS/PAH capsules by following the seed mediated approach as described by Murphy *et al*., with modifications in seed and ligand concentrations [32], [40]. First seed was synthesized inside PSS/PAH capsules by dispersing the capsules in 1 ml of aqueous solution containing 0.25 mM tri sodium citrate and 0.25 mM chloroauric acid, followed by gentle mixing for 15 minutes. Freshly prepared ice cold 0.1 (M) sodium borohydride was added to the capsule solution drop wise while stirring and was left undisturbed for 2 hrs so as to form gold seeds inside capsules. Then, 900 µl of supernatant was removed by centrifuging at 5000 rpm for 4 min and the remaining solution containing Au nanoparticles loaded capsules was used as stock seed solution.

For synthesis of gold nanorods, these seed loaded PSS/PAH capsules (100 µl) were added in 40 ml of growth solution (containing 0.25 mM chloroauric acid, 0.01 (M) cetyltrimethylammonium bromide (CTAB) and 200 µl of 0.1 (M) freshly prepared ascorbic acid solution). The color of the solution changed to reddish brown after 5 min and the stirring was stopped at that point of time. After 20 min, the nanoparticles-loaded capsules were separated by centrifugation at 5000 rpm for 4 minutes followed by washing with water and redispersed in water.

For synthesis of gold nanoprisms, similar protocol as above was employed but the seed concentration was reduced 2.5 times. For synthesis of multifaceted gold nanostructures, same steps as in the above protocol were repeated but CTAB concentration used was 0.1(M) instead of 0.01(M).

### Co-loading of Au and LaVO_4_:Eu^3+^(5%) Nanoparticles in PSS/PAH Capsules

LaVO_4_:Eu^3+^(5%) nanoparticles were first synthesized in PSS/PAH capsules as per the protocol given above. These LaVO_4_:Eu^3+^(5%) nanoparticles-loaded capsules were then washed thrice and Au nanoparticles were prepared in them by the same method as described for loading of gold nanoparticles in PSS/PAH capsules.

### Co-loading of Au Nanoparticles and RITC-labeled Bovine Serum Albumin (BSA) in PSS/PAH Capsules

First PSS/PAH capsules were loaded with RITC-labeled BSA (BSA was labeled with RITC as per the same method as used for RITC-PAH above) as per the following protocol. 10 mg APTS-MS was incubated with 0.5 ml of RITC-BSA solution (1 mg/ml in DW) for ∼30 h with gentle mixing. The above particles were then coated with five bilayers of PSS/PAH. The silica core was then etched by using 5 M HF and the resulting RITC-BSA loaded capsules were washed five times with distilled water. These RITC-BSA loaded capsules were then used for loading of gold nanoparticles by the same method as described for loading of gold nanoparticles in PSS/PAH capsules.

### Cell Uptake Studies

HeLa cells were cultured in DMEM medium containing heat inactivated FBS (10% v/v) and penicillin/streptomycin (1% v/v), grown at 37°C in a humidified atmosphere containing 5% CO_2_.

To investigate the uptake kinetics as a function of incubation time of nanoparticles-loaded capsules, HeLa cells were treated with LaF_3_:Tb^3+^ nanoparticles-loaded PSS/PAH capsules for different durations (2 h, 5 h, and 8 h; treatment ratio was 50 capsules (nanoparticles-loaded) per seeded cell; capsules were labeled with RITC-PAH). After the respective treatment, media was discarded and cells were washed thrice with PBS buffer (pH 7.4) to remove loosely bound capsules to the cells. Post washing, these cells were trypsinized by using 0.25% Trypsin-EDTA to further remove any capsules which have not yet internalized. The cells were then seeded on cover slips and left for few hours. After the cells adhered, they were fixed (and stained with FITC-Phalloidin) and imaged by fluorescence microscopy and scanning electron microscopy.

To determine cellular uptake of the nanoparticles-loaded capsules, HeLa cells (10^5^) were seeded on glass cover slip (13 mm, 0.2% gelatin coated) for 20 hours. The cells were then treated with LaF_3_:Tb^3+^ and Au nanoparticles-loaded PSS/PAH capsules (capsules were labeled with RITC-PAH, treatment ratio was 50 capsules (nanoparticles-loaded) per seeded cell) for 16 h. Further, the cells were washed; actin cytoskeleton of the cells was stained with FITC-Phalloidin (green) and imaged by confocal laser scanning microscopy.

### Flow Cytometry Studies

HeLa cells (10^5^ cells per well) were grown as described above and the cells were incubated with blank PSS/PAH capsules and Au nanoparticles-loaded PSS/PAH capsules for 26 h (treatment ratio was kept same for both blank capsules and nanoparticles-loaded capsules i.e. 100 capsules per seeded cell). After the incubation the cells were washed with PBS buffer (pH 7.4) and trypsinized by using 0.25% Trypsin-EDTA. The cells were then resuspended in complete media and analyzed by flow cytometry. The experiments were done in triplicate.

### MTT Assay

*In vitro* cytotoxicity studies of blank PSS/PAH capsules, GdF_3_:Tb^3+^(5%), LaF_3_:Tb^3+^(5%), and Au nanoparticles-loaded PSS/PAH capsules with HeLa cells were investigated by MTT assay [41]. 10^5^ cells per well were seeded in a 24 well plate. The cells were grown for 18 h, followed by changing it with media containing respective capsules (100 capsules (nanoparticles-loaded) per seeded cell) and blank capsules (100 capsules per seeded cell). In the control wells (i.e. only cells), media was added without capsules. The cells were incubated with the capsules for ∼22 h in a 37°C, 5% CO_2_ humidified incubator followed by removal of media. 500 µL of basal media having MTT (0.5 mg/ml MTT) was added to the wells and allowed to incubate for 4 h. Further, media containing MTT was removed from the wells and 1 ml of dimethyl sulphoxide (DMSO) was added into each well. The blue color solution from the wells was transformed into a cuvette and absorbance at 570 nm was measured. All the assays were done in triplicate.

### Characterization

UV-Vis spectra were recorded from UV-1800 Shimadzu UV spectrometer. The photoluminescence spectra and decay curves were recorded using Edinburgh instruments FLSP 920 fluorescence system. Emission and lifetime analyses of nanoparticles-loaded polymer capsules were done by exciting the samples with an Nd:YAG laser, attached with an optical parametric oscillator (OPO) with an optical range from 210–2400 nm. 450 W steady state Xe lamp was used to record the photoluminescence spectra of bare nanoparticles. The detector used was a red-sensitive Peltier element cooled Hamamatsu R928-P PMT. All the TEM images were obtained from FEI Technai G^2^ U-Twin (200 KeV) instrument. The size and surface morphology of nanoparticles-loaded capsules and blank capsules were characterized by Scanning Electron Microscope (SUPRA 40 VP Gemini, Zeiss, Germany). The Confocal laser scanning microscopy images of cells were obtained from Leica PCS SP5 confocal microscope (40x, oil objective). Flow cytometry measurements were done by using Partec CyFlow® space cell scanner. Number of blank PSS/PAH capsules and PSS/PAH capsules (loaded with nanoparticles) were quantified by flow cytometry.

## Supporting Information

Figure S1 **UV-Vis absorption spectra of Au and Ag bare nanoparticles.**
(TIF)

Figure S2 **Lifetime of lanthanide-doped nanoparticles-loaded polymer capsules.** Decay curve of a) LaVO_4_:Eu^3+^(5%), b) LaF_3_:Tb^3+^(5%), and c) GdF_3_:Tb^3+^(5%) nanoparticles-loaded PSS/PAH capsule. The emission was monitored at 541 nm for Tb^3+^ doped sample and 612 nm for Eu^3+^ doped sample.
(TIF)

Figure S3 **Photoluminescence emission spectrum of blank PSS/PAH capsules.** Blank PSS/PAH capsules were subjected to photoluminescence spectroscopy so as to investigate the background emission contribution from the blank capsules.
(TIF)

Figure S4 **Photoluminescence emission spectra of bare nanoparticles.** Photoluminescence emission spectra of LaF_3_:Tb^3+^(5%), GdF_3_:Tb^3+^(5%), and LaVO_4_:Eu^3+^(5%) bare nanoparticles. The emission bands (green and red curve, Figure 1c) around 544, 584 and 619 nm are assigned to ^5^D_4_ to ^7^F_5_, ^7^F_4_, and ^7^F_3_ transitions, respectively of Tb^3+^ ions. The emission bands (black curve) around 591 nm (^5^D_0_ to ^7^F_1_), 615 nm (^5^D_0_ to ^7^F_2_), and 696 nm (^5^D_0_ to ^7^F_4_) are assigned to Eu^3+^ ions.
(TIF)

Figure S5 **UV-Vis absorption spectra of 5 µm PSS/PAH capsules loaded with (a) gold nanorods and (b) multifaceted gold nanoparticles.**
(TIF)

Figure S6 **Co-loading of drug and nanoparticles within PSS/PAH capsules.** Fluorescence microscopy image of PSS/PAH capsules (∼5 µm) co-loaded with RITC-labeled BSA and Au nanoparticles.
(TIF)

Figure S7 **Validation of internalization of nanoparticles-loaded capsules by HeLa cells.** Confocal laser scanning microscopy sections (XY) of HeLa cells after uptake of LaF_3_:Tb^3+^ nanoparticles-loaded PSS/PAH capsules at different Z positions; (a) basal, (b) inside and (c) apical of the cell, and (d) XY,YZ and XZ section of the cell. Scale bar is 20 µm.
(TIF)

Figure S8 **2D plots of the events recorded with FACS for uptake studies.** (a) HeLa cells, (b) HeLa cells after incubation with blank PSS/PAH (FITC-PAH) capsules, and (c) HeLa cells after incubation with Au nanoparticles-loaded PSS/PAH capsules (FITC-PAH).
(TIF)

Table S1 **Stock solutions for preparing the nanoparticles-loaded polymer capsules.**
(DOC)

Table S2 **Lifetime of lanthanide-doped nanoparticles-loaded polymer capsules.** Average lifetime of Tb^3+^ or Eu^3+^ ions for different types of nanoparticles inside PSS/PAH capsules.
(DOC)

Scheme S1 **Schematic representation for the synthesis of nanomaterials-loaded PEM capsules.** Nanoparticles were synthesized inside the microvolume of capsules by incubating the capsules with nanoparticles precursors and citrate as ligand. (Inset is digital photograph of A) Au, B) Ag, C) LaVO_4_:Eu^3+^(5%), D) LaF_3_:Tb^3+^(5%), E) CdS, and F) GdF_3_:Tb^3+^(5%) nanoparticles-loaded PSS/PAH capsules and G) blank PSS/PAH capsules dispersed in PBS buffer (pH∼7.2)).
(TIF)
