# Supplementary figures and images for: Generic Delivery of Payload of Nanoparticles Intracellularly via Hybrid Polymer Capsules for Bioimaging Applications
Source: PLoS One. 2012 May 23;7(5):e36195. doi: 10.1371/journal.pone.0036195 (PMC3359331; doi:10.1371/journal.pone.0036195)

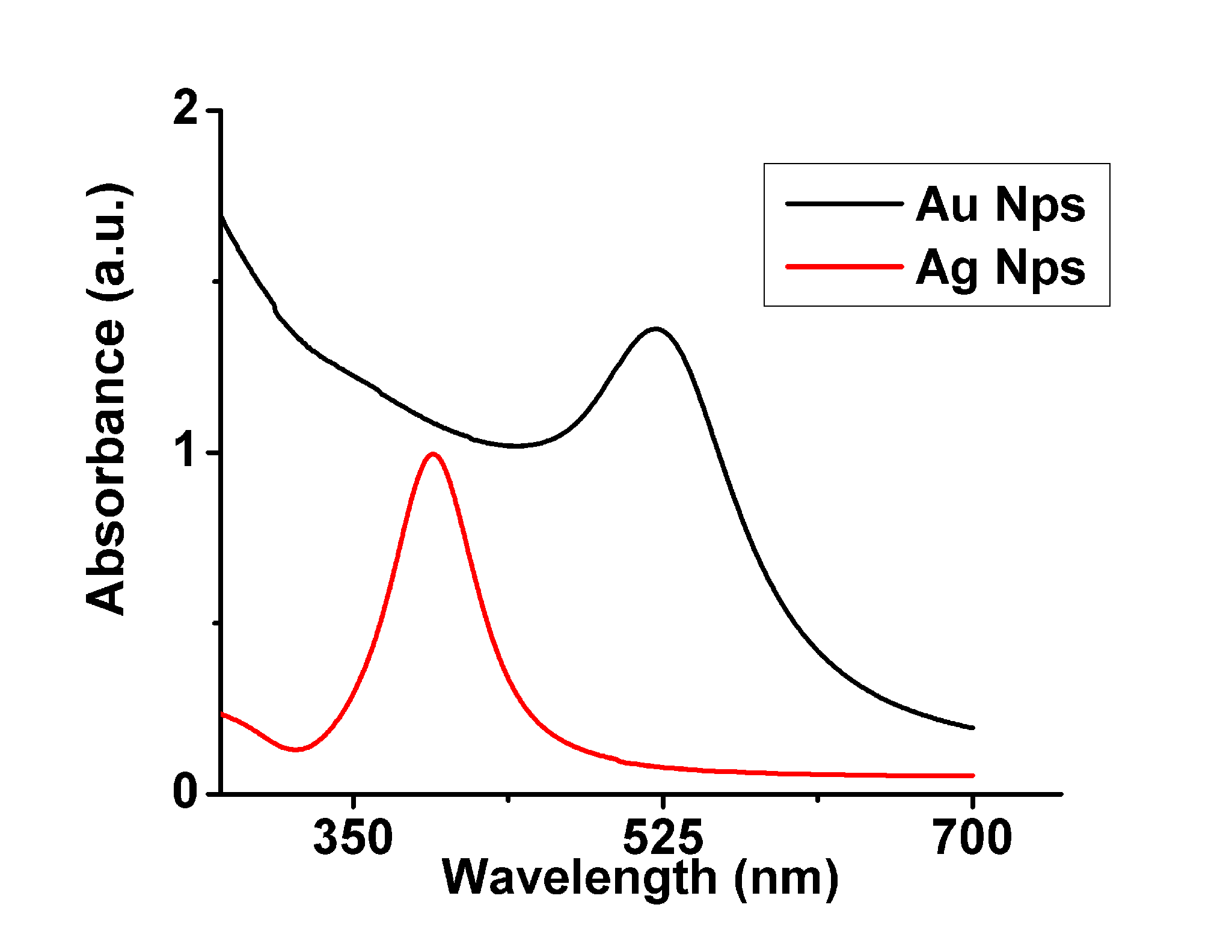

Supplement: Figure S1 — UV-Vis absorption spectra of Au and Ag bare nanoparticles. (TIF) [file pone.0036195.s001.tif]

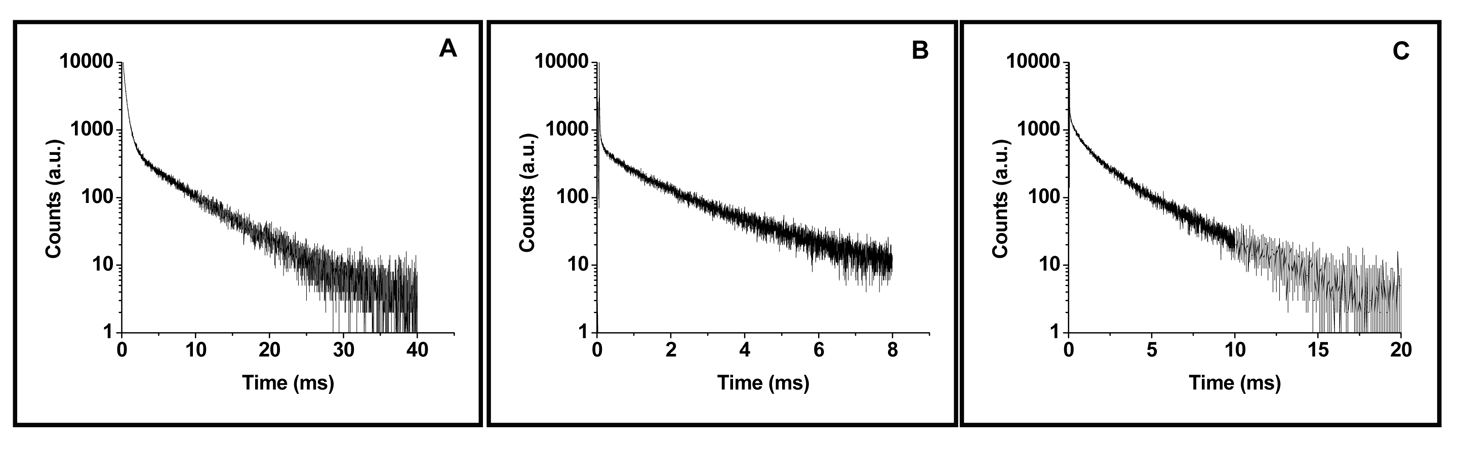

Supplement: Figure S2 — Lifetime of lanthanide-doped nanoparticles-loaded polymer capsules. Decay curve of a) LaVO4:Eu3+(5%), b) LaF3:Tb3+(5%), and c) GdF3:Tb3+(5%) nanoparticles-loaded PSS/PAH capsule. The emission was monitored at 541 nm for Tb3+ doped sample and 612 nm for Eu3+ doped sample. (TIF) [file pone.0036195.s002.tif]

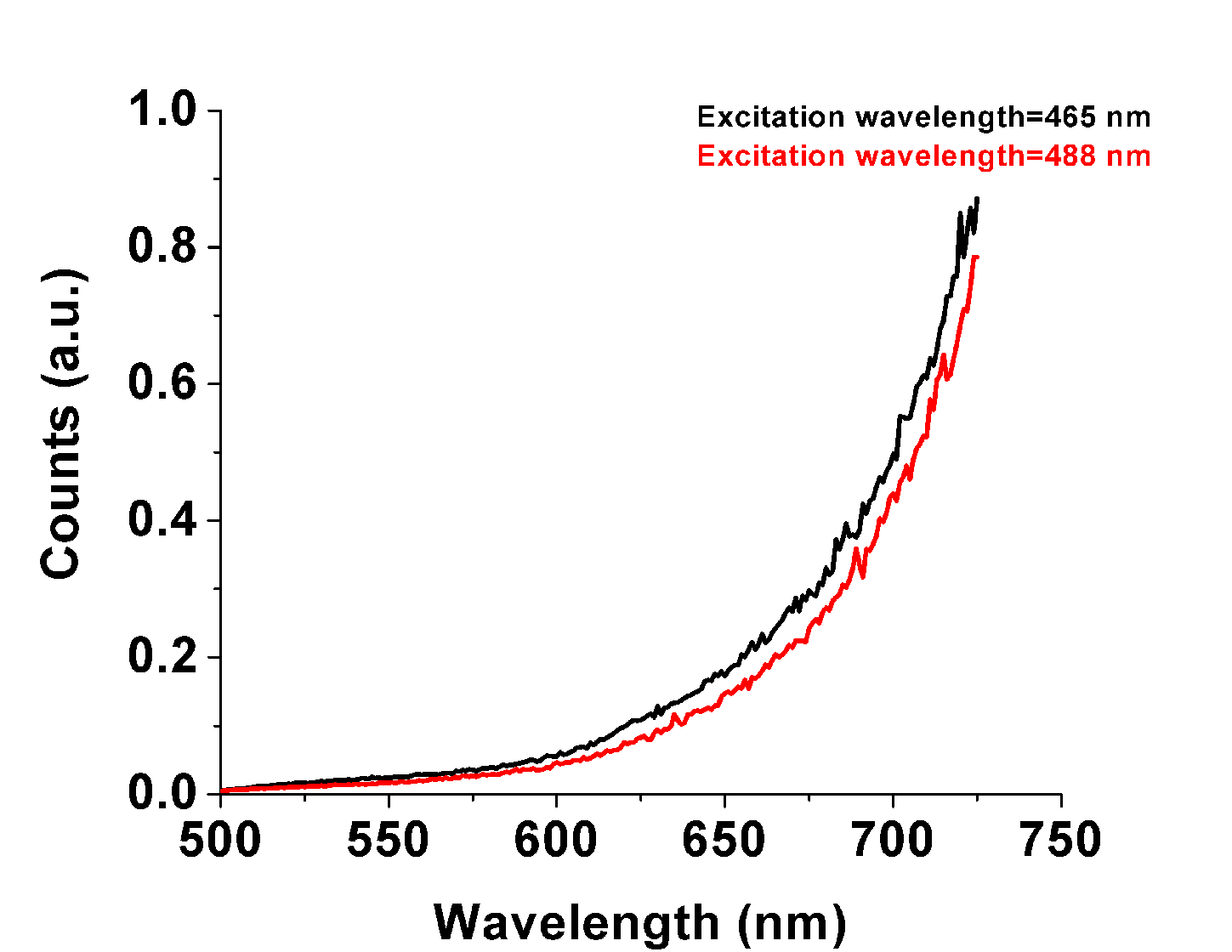

Supplement: Figure S3 — Photoluminescence emission spectrum of blank PSS/PAH capsules. Blank PSS/PAH capsules were subjected to photoluminescence spectroscopy so as to investigate the background emission contribution from the blank capsules. (TIF) [file pone.0036195.s003.tif]

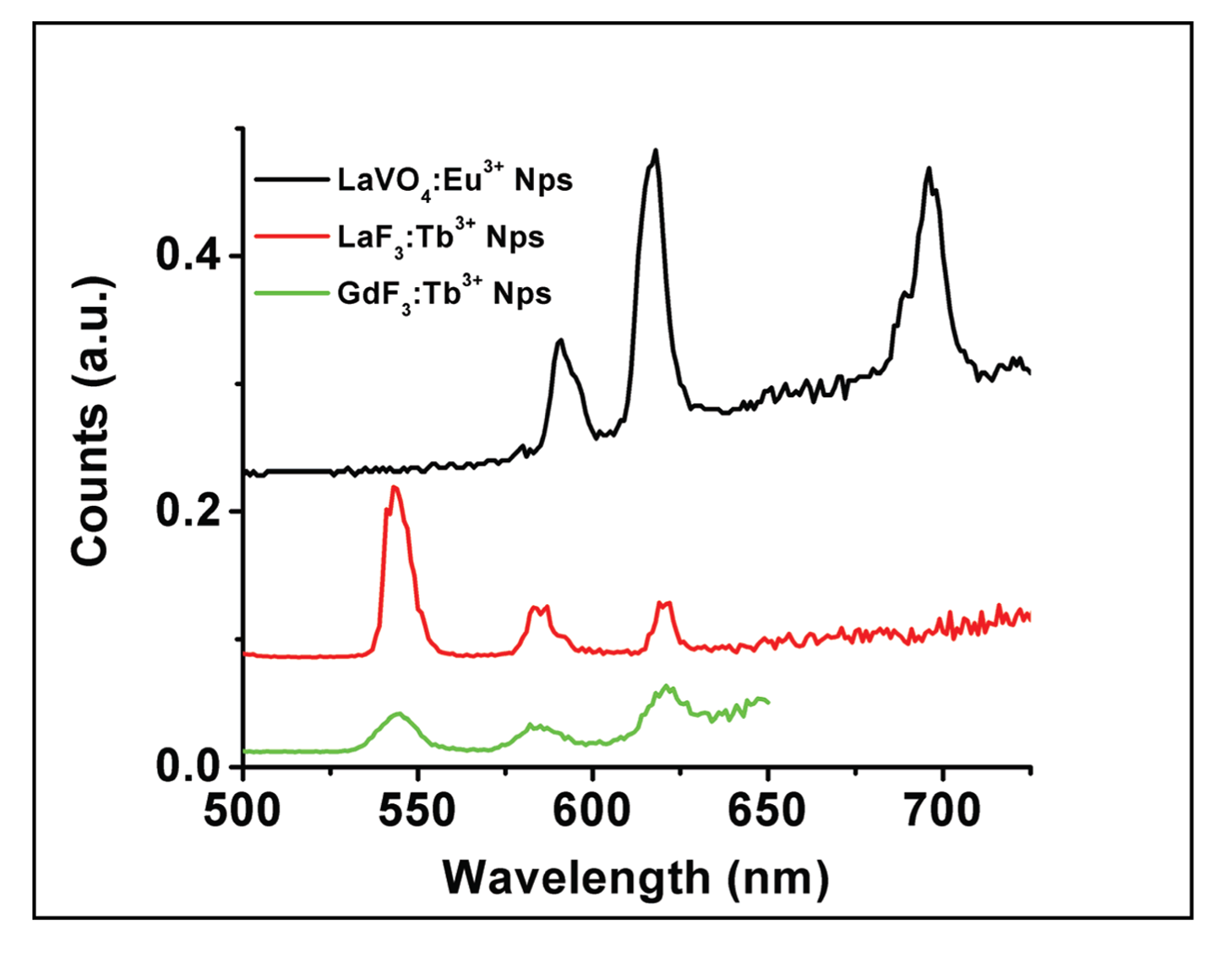

Supplement: Figure S4 — Photoluminescence emission spectra of bare nanoparticles. Photoluminescence emission spectra of LaF3:Tb3+(5%), GdF3:Tb3+(5%), and LaVO4:Eu3+(5%) bare nanoparticles. The emission bands (green and red curve, Figure 1c) around 544, 584 and 619 nm are assigned to 5D4 to 7F5, 7F4, and 7F3 transitions, respectively of Tb3+ ions. The emission bands (black curve) around 591 nm (5D0 to 7F1), 615 nm (5D0 to 7F2), and 696 nm (5D0 to 7F4) are assigned to Eu3+ ions. (TIF) [file pone.0036195.s004.tif]

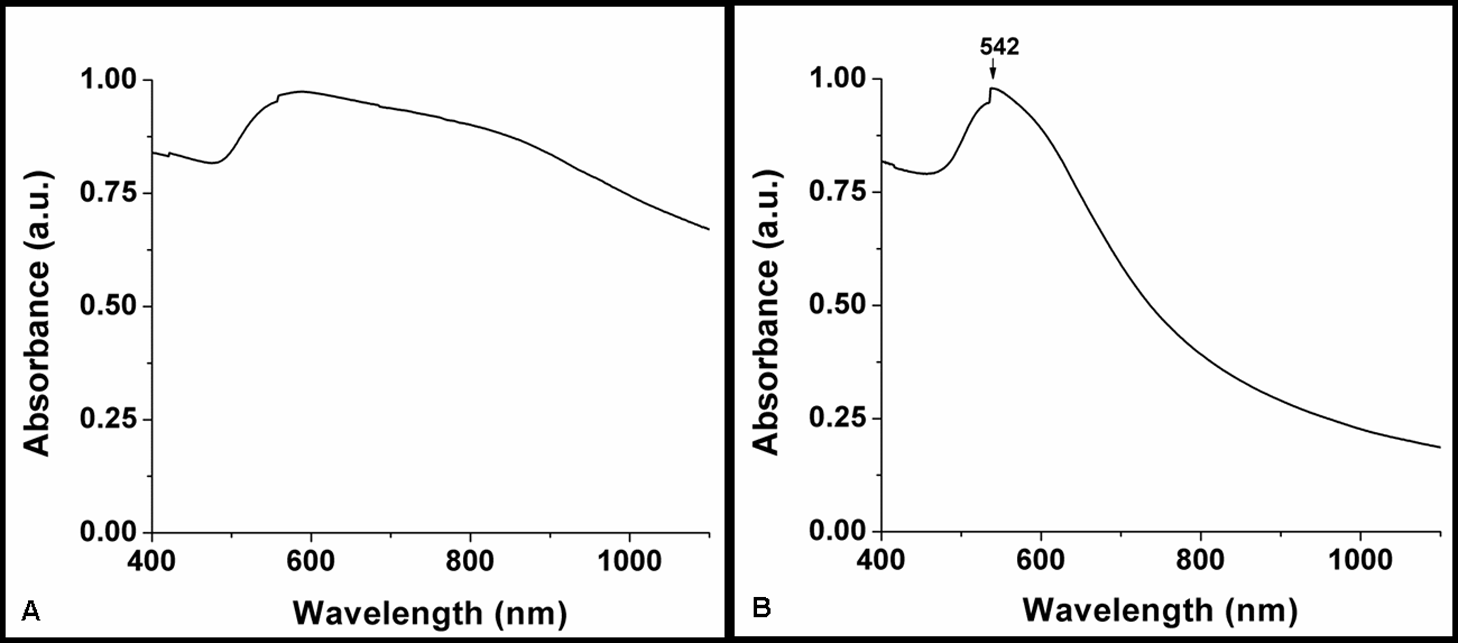

Supplement: Figure S5 — UV-Vis absorption spectra of 5 µm PSS/PAH capsules loaded with (a) gold nanorods and (b) multifaceted gold nanoparticles. (TIF) [file pone.0036195.s005.tif]

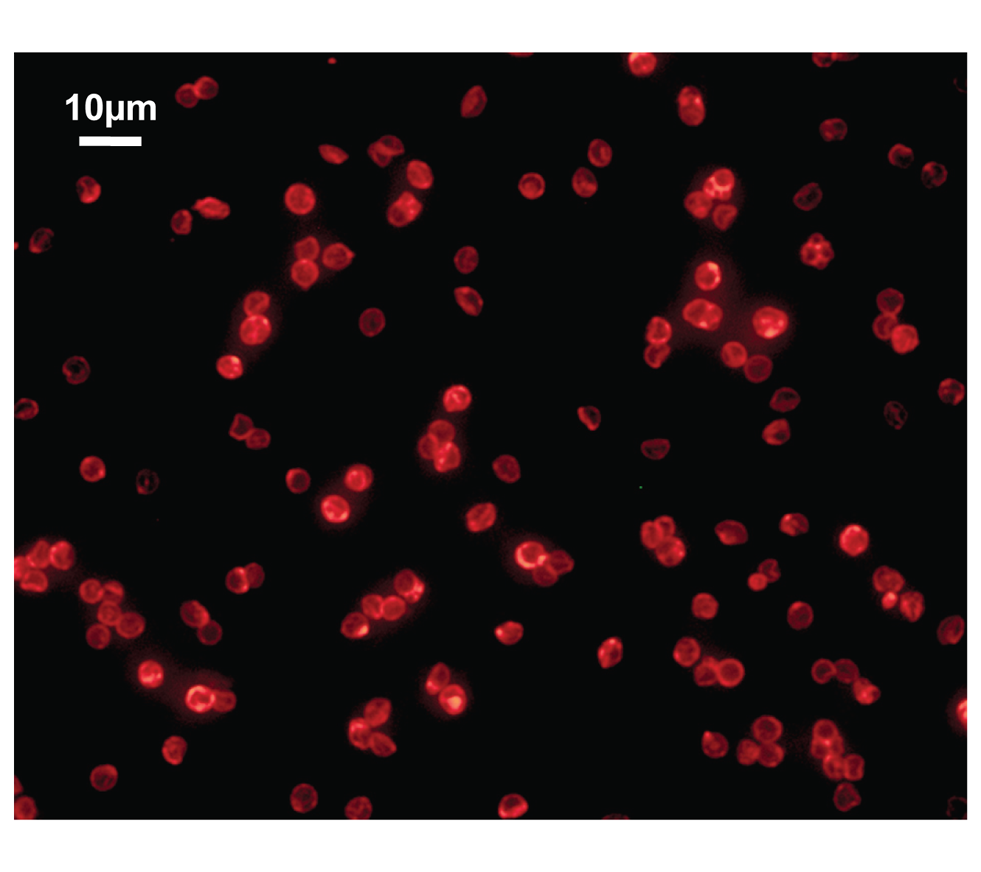

Supplement: Figure S6 — Co-loading of drug and nanoparticles within PSS/PAH capsules. Fluorescence microscopy image of PSS/PAH capsules (∼5 µm) co-loaded with RITC-labeled BSA and Au nanoparticles. (TIF) [file pone.0036195.s006.tif]

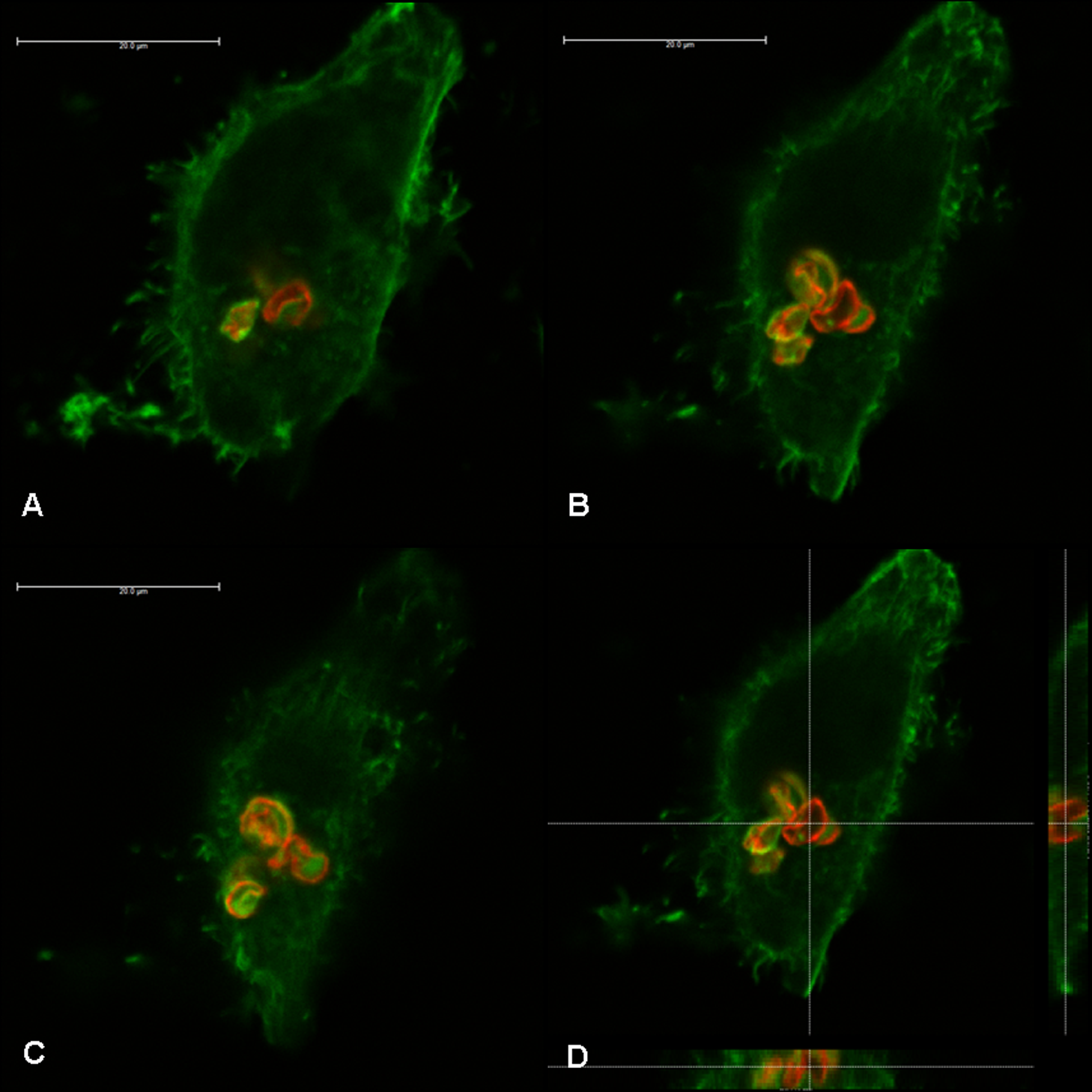

Supplement: Figure S7 — Validation of internalization of nanoparticles-loaded capsules by HeLa cells. Confocal laser scanning microscopy sections (XY) of HeLa cells after uptake of LaF3:Tb3+ nanoparticles-loaded PSS/PAH capsules at different Z positions; (a) basal, (b) inside and (c) apical of the cell, and (d) XY,YZ and XZ section of the cell. Scale bar is 20 µm. (TIF) [file pone.0036195.s007.tif]

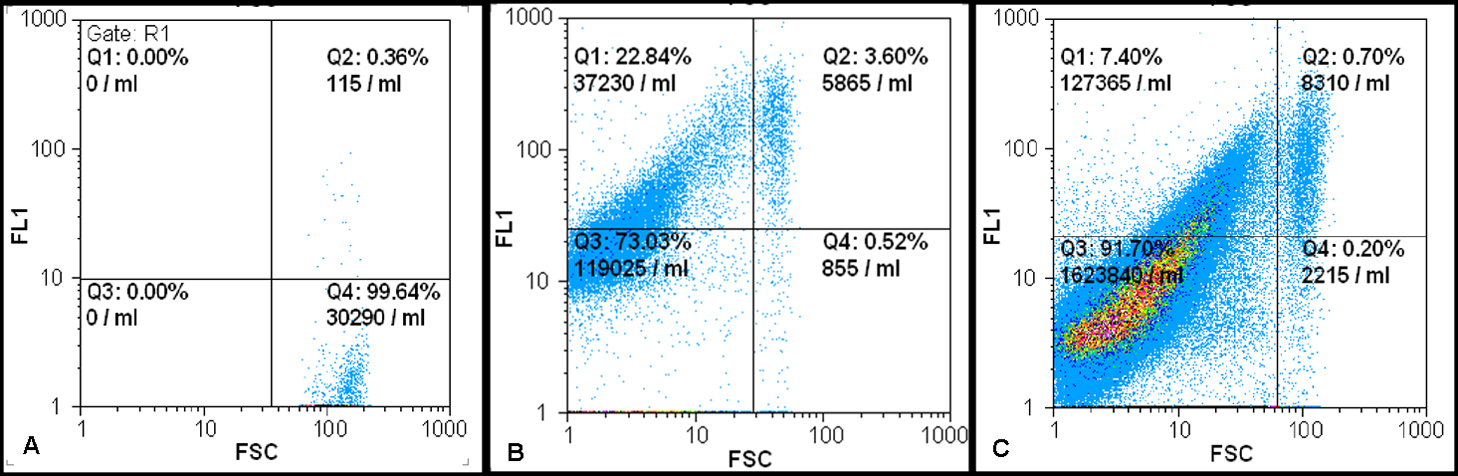

Supplement: Figure S8 — 2D plots of the events recorded with FACS for uptake studies. (a) HeLa cells, (b) HeLa cells after incubation with blank PSS/PAH (FITC-PAH) capsules, and (c) HeLa cells after incubation with Au nanoparticles-loaded PSS/PAH capsules (FITC-PAH). (TIF) [file pone.0036195.s008.tif]

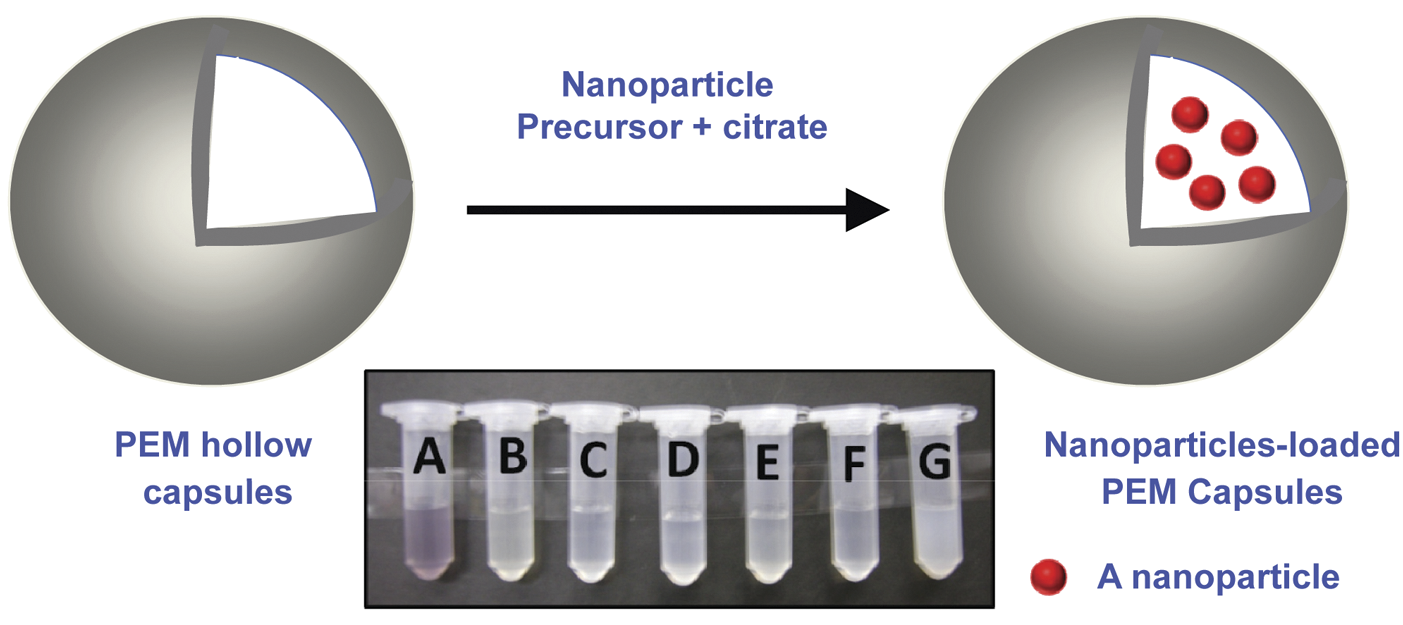

Supplement: Scheme S1 — Schematic representation for the synthesis of nanomaterials-loaded PEM capsules. Nanoparticles were synthesized inside the microvolume of capsules by incubating the capsules with nanoparticles precursors and citrate as ligand. (Inset is digital photograph of A) Au, B) Ag, C) LaVO4:Eu3+(5%), D) LaF3:Tb3+(5%), E) CdS, and F) GdF3:Tb3+(5%) nanoparticles-loaded PSS/PAH capsules and G) blank PSS/PAH capsules dispersed in PBS buffer (pH∼7.2)). (TIF) [file pone.0036195.s011.tif]
