# Supplementary material for: Generic Delivery of Payload of Nanoparticles Intracellularly via Hybrid Polymer Capsules for Bioimaging Applications
Source: PLoS One. 2012 May 23;7(5):e36195. doi: 10.1371/journal.pone.0036195 (PMC3359331; doi:10.1371/journal.pone.0036195)
Supplement: Table S1 — Stock solutions for preparing the nanoparticles-loaded polymer capsules. (DOC) [file pone.0036195.s009.doc]

**Table S1.** Stock solutions for preparing the nanoparticles-loaded polymer capsules

| **Sample** | **Stock solution A** | **Stock solution B** | **Stock solution C** |
| --- | --- | --- | --- |
| Au-loaded PSS/PAH | 1 mL HAuCl_4_ (1mM) | 100 μL trisodium citrate (38.8 mM) | 2 μL NaBH_4_ (0.1M) |
| Ag-loaded PSS/PAH | 0.9 mL AgNO_3_ (0.1 mM) | 100 μL trisodium citrate (0.8 mM) | 100 μL NaBH_4_ (2mM) |
| CdS-loaded PSS/PAH | 1 mL Cd(NO_3_)_2_ (10 mM) | 6 μL sodium citrate (34 mM) | 56 μL Na_2_S (8mM) |
| LaF_3_:Tb^3+^(5%)-loaded PSS/PAH | 900 µL citric acid in water (0.26 M) and pH ~6 was maintained by added NH_4_OH (25% w/w) | 50 μL methanol containing La(NO_3_)_3_.xH_2_O (0.5 mM) + Tb(NO_3_)_3_.5H_2_O (0.028 mM) | 100 µL NaF in water (0.75 M) |
| GdF_3_:Tb^3+^(5%)-loaded PSS/PAH | 900 µl citric acid (0.26 M) and pH ~6 was maintained by added NH_4_OH (25% w/w) | 50 μL methanol containing Gd(NO_3_)_3_.6H_2_O (0.5 mM) + Tb(NO_3_)_3_.5H_2_O (0.028 mM) | 100 µl NaF in water (0.75M) |
| LaVO_4_:Eu^3+^(5%)-loaded PSS/PAH | 900 µl citric acid (0.26 M) and pH ~6 was maintained by added NH_4_OH (25% w/w) | 50 μL methanol containing La(NO_3_)_3_.xH_2_O (0.5 mM) and Eu(NO_3_)_3_.5H_2_O (0.028mM) | 100 µl Na_3_VO_4_ in water (0.125 M) |
