# Supplementary material for: Generic Delivery of Payload of Nanoparticles Intracellularly via Hybrid Polymer Capsules for Bioimaging Applications
Source: PLoS One. 2012 May 23;7(5):e36195. doi: 10.1371/journal.pone.0036195 (PMC3359331; doi:10.1371/journal.pone.0036195)
Supplement: Table S2 — Lifetime of lanthanide-doped nanoparticles-loaded polymer capsules. Average lifetime of Tb3+ or Eu3+ ions for different types of nanoparticles inside PSS/PAH capsules. (DOC) [file pone.0036195.s010.doc]

**Table S2.** Average lifetime of Tb^3+^ or Eu^3+^ ions for different types of nanoparticles inside PSS/PAH capsules.
